# Genome-Wide Association Study Identified CNP12587 Region Underlying Height Variation in Chinese Females

**DOI:** 10.1371/journal.pone.0044292

**Published:** 2012-09-05

**Authors:** Yin-Ping Zhang, Fei-Yan Deng, Tie-Lin Yang, Feng Zhang, Xiang-Ding Chen, Hui Shen, Xue-Zheng Zhu, Qing Tian, Hong-Wen Deng

**Affiliations:** 1 Key Laboratory of Environment and Genes Related to Diseases, Ministry of Education, College of Medicine, Xi’an Jiaotong University, Xi’an, P. R. China; 2 Department of Biostatistics and Bioinformatics, Tulane University School of Public Health and Tropical Medicine, New Orleans, Louisiana, United States of America; 3 The Key Laboratory of Biomedical Information Engineering, Ministry of Education and Institute of Molecular Genetics, School of Life Science and Technology, Xi’an Jiaotong University, Xi’an, P. R. China; 4 Laboratory of Molecular and Statistical Genetics, College of Life Sciences, Hunan Normal University, Changsha, P. R. China; 5 Center of Systematic Biomedical Research, Shanghai University of Science and Technology, Shanghai, P. R. China; National Taiwan University, Taiwan

## Abstract

**Introduction:**

Human height is a highly heritable trait considered as an important factor for health. There has been limited success in identifying the genetic factors underlying height variation. We aim to identify sequence variants associated with adult height by a genome-wide association study of copy number variants (CNVs) in Chinese.

**Methods:**

Genome-wide CNV association analyses were conducted in 1,625 unrelated Chinese adults and sex specific subgroup for height variation, respectively. Height was measured with a stadiometer. Affymetrix SNP6.0 genotyping platform was used to identify copy number polymorphisms (CNPs). We constructed a genomic map containing 1,009 CNPs in Chinese individuals and performed a genome-wide association study of CNPs with height.

**Results:**

We detected 10 significant association signals for height (*p*<0.05) in the whole population, 9 and 11 association signals for Chinese female and male population, respectively. A copy number polymorphism (CNP12587, chr18:54081842-54086942, *p* = 2.41×10^−4^) was found to be significantly associated with height variation in Chinese females even after strict Bonferroni correction (*p* = 0.048). Confirmatory real time PCR experiments lent further support for CNV validation. Compared to female subjects with two copies of the CNP, carriers of three copies had an average of 8.1% decrease in height. An important candidate gene, ubiquitin-protein ligase NEDD4-like (NEDD4L), was detected at this region, which plays important roles in bone metabolism by binding to bone formation regulators.

**Conclusions:**

Our findings suggest the important genetic variants underlying height variation in Chinese.

## Introduction

Height is an important physical index to reflect the processes of growth and development in clinical practice [1]. Variation of height is associated with a range of diseases, such as various cancers [2], type 2 diabetes [3], coronary heart disease [4]. Among the most visible traits that can be measured easily and accurately [5], adult human height is mainly influenced by genetic and environmental factors [6]. Genetic variation explains up to 90% of variation [7], [8], [9], specifically more than 60% in Han Chinese [10]. Therefore, a better understanding of the genetic variants underlying height difference might also provide novel insights into the clinical practice [11]. Previous investigations, including recent genome-wide association studies [6], [12], [13], [14], [15], [16] have discovered several genetic factors associated with height variation. However, all of these implicated genes or SNPs account for no more than 10% of the population variation in height. The majority of genetic variation accounting for adult height has not been determined yet.

**Table 1:** Basic characteristics of the study subjects.

Trait	Total (N = 1625)	Female (N = 823)	Male (N = 802)
Age (year)	34.49 (13.24)	37.45 (13.79)	31.43 (11.93)
Height (cm)	164.25 (8.16)	158.38 (5.22)	170.27 (5.96)
Weight (kg)	60.12 (10.48)	54.63 (8.09)	65.75 (9.64)
BMI (kg/cm^2^)	22.21 (3.03)	21.78 (3.05)	22.66 (2.93)

The presented data are mean (SD) of raw values.

Copy-number variations (CNVs) are now known to be widespread across human genome and functionally significant, accounting for nearly 20% of the total detected variation in gene expression [17]. CNVs range from one kilobase (Kb) to several megabases (Mb) with variations in the size of DNA fragments. Copy number polymorphisms (CNPs) refer to common CNVs that appear to involve the same affected genomic sequence and are therefore consistent with a model of a genetic polymorphism. As a common type of genomic variability, CNVs may include duplications or deletions [18], [19]. They can influence gene expression by disrupting coding sequences, perturbing long-range gene regulation, or altering gene dosage, and these effects could contribute to phenotypic variations [20] or disease risk [21], [22]. A number of studies have successfully identified CNVs related to complex human diseases, such as AIDS [23], immunologically mediated glomerulonephritis [24],Crohn disease [25] and neuroblastoma [26]. Recently, our groups performed three genome-wide CNV association studies, and found CNV regions containing UGT2B17 [21] and VPS13B [22] genes were significantly associated with BMD, and FHL2 gene with hip bone size [27].

To search for more genetic factors influencing adult height, we performed genome wide CNV analyses in a population of Chinese using Affymetrix Human Mapping 600K Arrays, which are effective in identification of genomic CNVs [28], [29]. For those CNPs that were significantly associated with height, we performed further analysis using real-time quantitative PCR to validate. Our findings support the importance of CNPs in the height variation of Chinese population.

**Figure 1:**
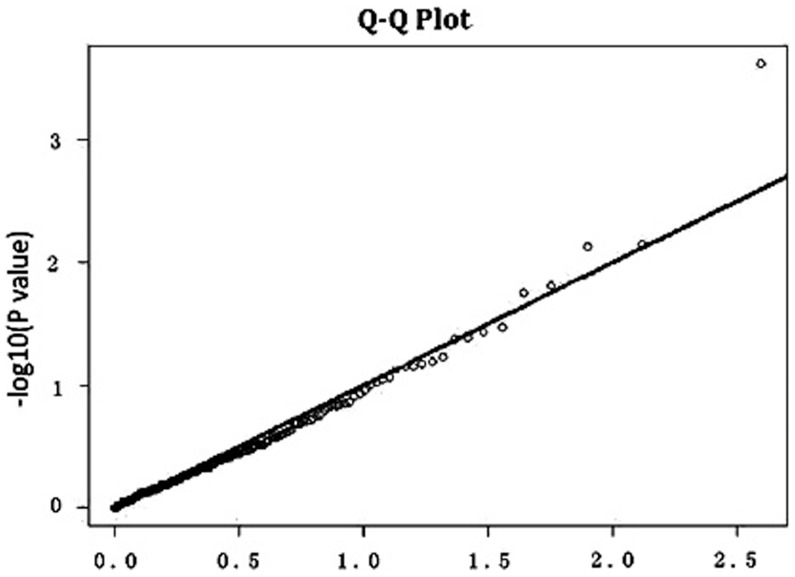
Quantile–quantile (Q–Q) plot for height. From the Q–Q plot, the observed *p*-values for height match the expected *p* -values under the null distributions over the range of (1<-log10(*p*)<3.0). Furthermore, an excess of low *p* -value is observed above 3.0 of -log10(*p*) for height.

**Table 2:** CNPs associated with height in the Chinese population (*p*<0.05).

Population (n)	NAME	Chr.	Start (bp)	End (bp)	Frequency AA (%)	QC value	P-value
Total (1625)	CNP363	3	1658250	1666567	93.99	0.024	4.21×10^−3^
	CNP11171	6	169249708	169260864	92.26	0.002	4.89×10^−3^
	CNP12439	16	34879419	34916829	98.51	0.011	4.95×10^−3^
	CNP2062	15	22226226	22269689	55.85	0.043	1.34×10^−2^
	CNP1679	11	5744656	5765715	60.57	0.049	1.97×10^−2^
	CNP1452	9	67675871	67678367	29.71	0.048	2.84×10^−2^
	CNP11937	11	134119190	134132097	98.03	0.040	2.97×10^−2^
	CNP1162	7	133435735	133449694	59.47	0.024	3.94×10^−2^
	CNP1811	12	9524645	9619559	32.21	0.002	4.33×10^−2^
	CNP1236	8	5586134	5591735	90.11	0.007	4.46×10^−2^
Female (823)	***CNP12587***	***18***	***54081842***	***54086942***	***98.82***	***0.043***	***2.41*** **×** ***10*** **^−^** ^***4***^
	CNP1162	7	133435735	133449694	60.63	0.024	7.04×10^−3^
	CNP1175	7	141693868	141712586	11.72	0.002	7.36×10^−3^
	CNP11171	6	169249708	169260864	90.82	0.002	1.55×10^−2^
	CNP603	4	69043083	69168574	0.87	0.037	1.75×10^−2^
	CNP1726	11	49716131	49717264	70.36	0.039	3.37×10^−2^
	CNP11858	11	34177080	34179954	98.40	0.033	3.66×10^−2^
	CNP2406	19	40541333	40553688	54.06	0.027	4.14×10^−2^
	CNP2007	14	40680246	40727099	74.13	0.040	4.22×10^−2^
Male (802)	CNP10211	1	223725840	223750819	98.78	0.019	9.10×10^−4^
	CNP12439	16	34879419	34916829	98.94	0.011	1.50×10^−3^
	CNP1119	7	90869671	90878663	64.29	0.021	2.02×10^−2^
	CNP11043	6	31467630	31559455	95.90	0.015	2.21×10^−2^
	CNP12091	13	63227094	63303323	98.03	0.018	2.79×10^−2^
	CNP1675	11	4924689	4933658	43.01	0.034	2.89×10^−2^
	CNP363	3	1658250	1666567	94.17	0.024	3.44×10^−2^
	CNP10849	4	178443731	178452258	98.33	0.016	3.62×10^−2^
	CNP1236	8	5586134	5591735	89.89	0.007	4.14×10^−2^
	CNP2736	24	24720477	25412124	29.68	0.015	4.72×10^−2^
	CNP12061	13	21994827	22004855	97.35	0.040	4.84×10^−2^

**Note:** The italatic and bond represents the significant CNP after Bonferroni correction.

## Materials and Methods

### Subjects

The study was approved by the local institutional review boards and the office of research administration of participating institutions. After signing an informed consent, all subjects completed a structured questionnaire on anthropometric variables, lifestyle, and medical history.

This Genome-wide association study sample contained 1,625 unrelated Chinese Han adults, including 823 women and 802 men. The samples were randomly identified from our established and expanding database currently containing more than 6,000 subjects. All subjects were healthy subjects defined by a comprehensive suite of exclusion criteria [30]. Briefly, subjects with chronic diseases and conditions involving vital organs (heart, lung, liver, kidney, and brain) and severe endocrinological, metabolic, and nutritional diseases that might affect human development were excluded from this study. The purpose is to minimize the confounding effects of environmental and therapeutic factors which may interfere with association test and increase the power of detecting modest genetic effect on height variation in our study population. Height was measured using a calibrated stadiometer. The basic characteristics of the study sample sets are summarized in Table 1.

### Genotyping

Genomic DNA was extracted from peripheral blood leukocytes using standard protocols. Genome-Wide Human SNP Array 6.0 (Affymetrix, Santa Clara, CA, USA), which features 1.8 million genetic markers, including more than 906,600 SNPs and more than 946,000 probes for detection of copy number variation, was performed using the standard protocol recommended by the manufacturer. Fluorescence intensities were quantified using an Affymetrix array scanner 30007G. Data management and analyses were performed using the Affymetrix GeneChip Command Console Software (AGCC). Contrast quality control (QC) threshold was set at the default value of greater than 0.4 for sample quality control. The final average contrast QC across the entire sample reached the high level of 2.62. The Birdsuite package (http://www.broadinstitute.org/science/programs/medical-and-population-genetics/birdsuite/birdsuite-0) was used for genotype calling, genotyping quality control, and CNV identification.

For the QC for sample, we firstly measured the copy number estimates of each chromosome and genome-wide average (sum of all chromosomes), reported by the Birdseye Hidden Markov Model [31], and removed the subjects who showed excessively high or low estimate for copy number according to either genome-wide average or more than 2 chromosomes (>3 standard deviations). Then, we measured the variability of CNP and SNP probe intensities according to each chromosome and genome-wide average (sum of all chromosomes). We removed the subjects with excessive variability in probe intensity according to either genome-wide average or more than 2 chromosomes (>3 standard deviations). We kept the subjects who only had 1 or 2 chromosomes failing in copy number estimate QC and probe intensity QC, and treated the CNPs in the chromosomes of those subjects as missing data in further association analysis. As a result, 1,531 samples were used in CANARY software [31] for CNP call.

**Figure 2:**
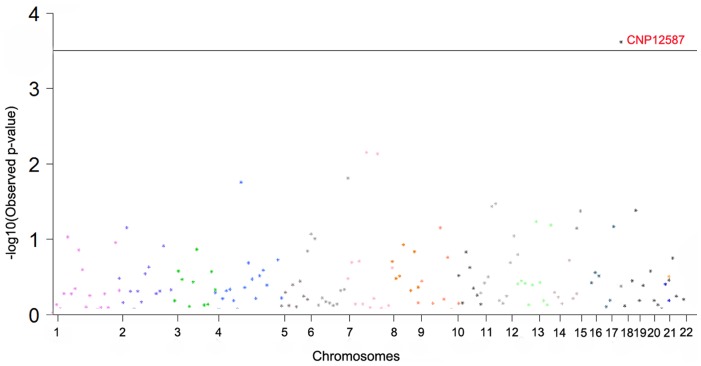
Genome-wide CNP association signals for height in Chinese females. Manhattan plot of the *p*-values (-Log10(Observed *p*-value)) from the height genome-wide association analysis. Each color identifies an autosomal chromosome (from chromosome 1 to chromosome 22). The horizontal line displays the cutoff for genome significance level after strict Bonferroni correction. The spacing between the CNPs does not reflect the actual distances between CNPs in the genome.

**Figure 3:**
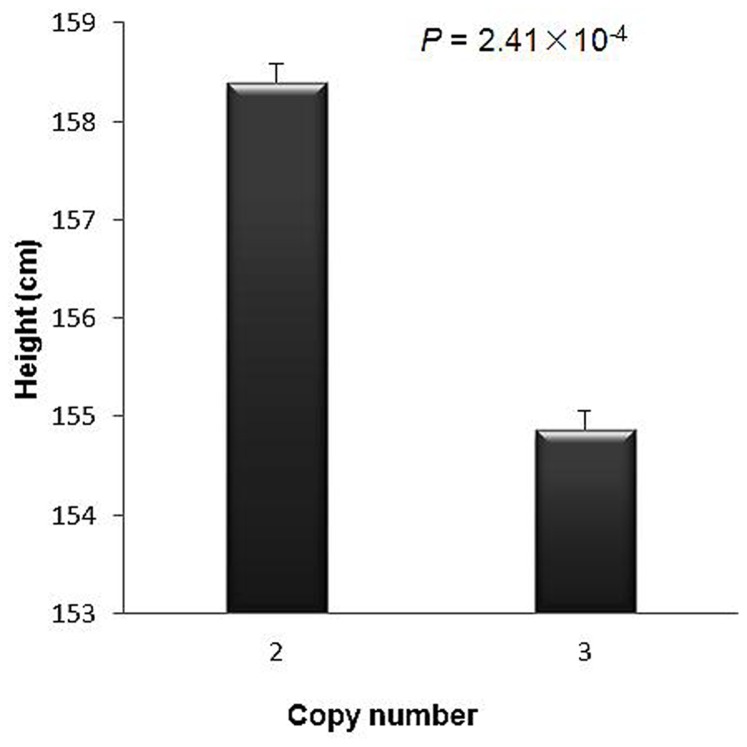
Difference in height between Chinese female carriers of two vs. three copies at CNP12587. 2 and 3 stands for normal and gain state, respectively.

For the QC for CNPs, we discarded: 1) any CNPs where more than 5% of the copy calls were uncertain (confidence score >0.1) or missing; 2) any CNPs with the frequency less than 1%. As a result, 198 CNPs out of the initial full-set of 1,009 CNPs were available for subsequent association analyses.

### Statistical Analyses

We used stepwise regression model to screen significant covariates. Parameters including age, age^2^, sex, age-sex, age^2^-sex, weight, BMI, birth year were tested for their association with height. Significant (*p* < 0.05) parameters (age, sex) were then included as covariates to adjust the raw height values. EIGENSTRAT was employed to perform principal component analysis to correct for stratification in genome-wide association studies [32]. We used 370,000 SNPs to calculate the principal components and the ten default main eigenvectors were used as covariates to adjust the raw height values for correction of population stratification. The adjusted height data, if not following normal distributions, were further subjected to BoxCox transformation into normal distribution. Finally, association analyses between CNPs and height data were performed using the PLINK software package (version 1.07) (http://pngu.mgh.harvard.edu/,purcell/plink/). Analyses of variance (ANOVA) were performed. The independent variable was the CNP, which was divided into three levels according to the CN (gain, CN>2; normal, CN = 2; and loss, CN<2). A raw *p* value <0.05 was considered nominally significant, which was further subjected to a Bonferroni correction to account for multiple testing in this study, where a significance level of 0.05/N (N = 198, i.e., the total number of the tested CNPs) was used as a significance threshold for a test (*p* = 2.53×10^−4^). Considering that gender heterogeneity may have significant contribution to height variation [33], we further analyzed the CNP effect on height in Chinese males and females, respectively.

### Real-Time PCR

We selected all 12 subjects (One doesn’t have additional DNA sample in total 13 subjects.) predicted to have duplications, and 12 subjects predicted to be diploid variants to perform real-time quantitative PCR, in order to assess the statistical significance of differences in DNA-amplification rates between groups with different copy numbers. The amplification rate is highly correlated with the copy numbers at the CNV. The forward primer is 5′-CATGGATTGTCTCGGGAGTT-3′, and the reverse primer is 5′- ACAGGCAGCAGAAAGCATCT-3′. Reactions were conducted in a 96 plate with the ABI 7500HT Sequence Detector system (Applied Biosystems Inc.,USA). Amplicons were designed against the putative altered locus and a control locus (C10orf11), which was used for controlling differences in genomic-DNA purity and concentration of different samples. PCR was performed in a 20 µl reaction volume containing 10 µl SuperReal PreMix (containing SYBR Green) (TIANGEN, Biotech, Beijing, China), 10 pmol forward and reverse primers, and 125 ng of genomic DNA. The reaction cycling conditions were 95°C for 15 min, followed by 40 cycles at 95°C for 10 s and at 60°C for 32 s. Sequence Detection Software (SDS) was used for exporting the threshold cycle (Ct) data and further analyzing differences in Ct values (ΔCt) between the test locus and the control locus. For groups predicted to have different copy numbers, a *t* test, with the significant threshold defined by *p*<0.05, was used for comparing ΔCt values to determine the statistical significance of these predicted copy-number differences.

## Results

The basic characteristics of the 1,625 Chinese Han subjects are summarized in Table 1. They averaged 34.49±13.24 years in age, 164.25±8.16 cm in height. The EIGENSTRAT program revealed that all subjects in this Chinese sample were clustered together and could not be assigned into any subgroups, indicating that there was no significant population stratification within the sample. The relative homogeneity of this study sample eliminates potential spurious associations due to population stratification.

We further created a quantile–quantile (Q–Q) plot for the distribution of *p* values involving the 198 CNPs in our sample (Fig. 1). The observed *p* values for height matched the expected *p* values over the range of 1<−log10(*p*)<3.0. The departure was observed at the extreme tail (−log10(*p*)>3.5) of the distribution of test statistics for height, suggesting that the associations identified are likely due to true variants rather than potential biases such as genotyping error, sample relatedness or potential population stratification. Table 2 list association results in the total sample, females only, and males only (*p*<0.05). The prominent association signals (*p*<0.01) for height were observed for chromosome regions 3p26.3 (CNP363), 6q27 (CNP11171), 16p11.1 (CNP12439) in the whole population, for chromosome regions 18q21.31 (CNP12587), 7q33 (CNP1162), 7q34 (CNP1175) in the female subgroup, for chromosome regions 1q41 (CNP10211), 16p11.1 (CNP12439) in the male subgroup.

The most significant association was detected at CNP363 (3p26.3, *p* = 4.21×10^−3^) in the whole population, CNP12587 (18q21.31, *p* = 2.41×10^−4^) in the female subgroup, CNP10211 (1q41, *p* = 9.10×10^−4^) in the male subgroup, respectively. After stringent Bonferroni correction, the association signal CNP12587 from female subgroup remained significant (*p* = 0.048). CNP12587 was located in 18q21.31 with the physical position from 54 081 842 bp to 54 086 942 bp (Table 2). According to the UCSC Human Genome Browser (http://genome.ucsc.edu/cgi-bin/hgGateway), the ubiquitin-protein ligase NEDD4-like (NEDD4L) gene is the only gene overlapping with CNP12587. Figure 2 further illustrates the genome-wide association signals for height variation on all 22 autosomes in female subgroup.

For CNP12587, of the 823 analyzed female subjects, 13 were carriers of three copies, representing a minor copy number frequency of 1.58%. In the female sample, compared to two copies of CNP12587, individuals with three CNs have an average of 8.1% decrease in height (Figure 3). As shown in Table 2, the association of CNP12587 with height is gender-specific (*p*<0.05). To validate the association between the CNP12587 and height, we genotyped the CNP12587 copy number by real time PCR. Based on 2^−ΔΔ^Ct [34], we performed Student’s *t* test to confirm the differential CNP. The relative copy numbers from *q*RT-PCR was 0.456±0.111 (mean ± SD) in two CNs group and 1.067±0.123 in three CNs group for CNP12587, with a *p* value less than 0.001. Confirmatory real time PCR experiments lent further support for CNV validation.

## Discussion

CNV is a genetic polymorphism recently recognized to be associated with human complex trait, presumably via a dosage effect on gene expression. This study identified that CNP12587 (18q21.31) was significantly associated with height in Chinese females. Confirmatory real time PCR experiments lent further support for CNV validation. The only gene overlapping with CNP12587 is ubiquitin-protein ligase NEDD4-like (NEDD4L), implicating the gene as new susceptibility genes for height variation in Chinese females.

The NEDD4L gene is located on human chromosome 18q, which has long been investigated since partial deletions of the long arm of chromosome 18 lead to variable phenotypes, such as short height and developmental delay [35], [36], [37], [38]. In a genome-wide linkage analysis for adult height, 18q21-22 was among the four regions with LOD scores above 2.0, with a maximum LOD score of 3.12 [39]. The NEDD4L gene is a member of the HECT (Homologous to the E6-AP Carboxyl Terminus) class of E3 ubiquitin ligases. An E3 ubiquitin ligase (also called a ubiquitin ligase), in combination with an E2 ubiquitin-conjugating enzyme, causes the attachment of ubiquitin to a lysine on a target protein via an isopeptide bond [40]. Ubiquitination is involved in multiple cellular functions, including proteasomal degradation and the control of stability, function, and intracellular localization of a wide variety of proteins [41]. Ubiquitination of proteins, mediated by E3 ubiquitin ligase, controls numerous cellular processes [42]. Many Ubiquitin (Ub) protein ligases (E3s) target both their substrates and themselves for degradation [43].

Ubiquitin ligase NEDD4L, previously identified as a regulator of renal sodium channels, could target activated Smad2/3 to limit TGF-beta signaling [44]. TGF-beta, a secreted factor present at high levels in bone, inhibits osteoblast differentiation [45] and controls osteogenic differentiation [46]. As potent stimulators of bone formation, TGF-beta is also involved in the regulation of endochondral and intramembranous ossification during human bone development in vivo [47]. TGF-beta functions during embryogenesis and in adult organism [47]. It is likely that NEDD4L gene may exert its effect on height via TGF-beta signaling.

It is notable that all the subjects in our Chinese sample were of the same Han ethnicity. The homogeneity of our sample minimized or eliminated copy-number polymorphisms in ethnically diverse populations, or other factors caused by population stratification. It is important to recognize that estimation of raw copy numbers from SNP-mapping array data is based on the ratio of SNP probe-set signal intensity for each test sample versus a reference set. Thus, statistical software uses the average of the reference set to infer changes in copy number by relative duplication or deletion. A larger sample size for the reference set can improve the accuracy of copy-number computation [48]. Similarly, for a specific CNV, exclusion of subjects with homozygous deletions from the reference set can also improve the precision of copy-number inference, as a result, in part, of unbiased signal intensities of a normal reference set.

In summary, our genome-wide CNV association study for height variation in Chinese, strongly suggest that CNP12587 (NEDD4L gene) is the novel candidate loci (gene) for height variation in Chinese females.
